# A case of ovarian clear cell carcinoma arising from ovarian mature cystic teratoma

**DOI:** 10.1186/s13048-018-0446-4

**Published:** 2018-08-30

**Authors:** Kazuya Maeda, Yoshito Terai, Shinichi Terada, Hiroshi Maruoka, Yuhei Kogata, Keisuke Ashihara, Yoshimichi Tanaka, Tomohito Tanaka, Hiroshi Sasaki, Satoshi Tsunetoh, Takashi Yamada, Masahide Ohmichi

**Affiliations:** 1 Department of Obstetrics and Gynecology, Osaka Medical College, 2-7, Daigaku-machi, Takatsuki, Osaka, 569-8686 Japan; 2 Department of Pathology, Osaka Medical College, 2-7, Daigaku-machi, Takatsuki, Osaka, 569-8686 Japan

**Keywords:** Ovary, Clear cell carcinoma, Mature cystic teratoma, Malignant transformation

## Abstract

**Background:**

It is well known that ovarian mature cystic teratomas (MCTs) occasionally go through malignant transformations. Among these, approximately 75% of histological types are squamous cell carcinoma, with the other types being exceptionally rare. We report an extremely rare case of ovarian clear cell carcinoma arising from ovarian mature cystic teratoma.

**Case presentation:**

The case was a 71-year-old woman with abdominal distention. Ultrasonography and magnetic resonance imaging showed a huge mass in her abdominal cavity. Fluorodeoxyglucose-positron emission tomography (FDG-PET) showed FDG uptake not only in the pelvic tumor but also in the hepatic nodule, thus suggesting metastases. We performed a total abdominal hysterectomy, bilateral salpingo-oophorectomy, and an omentectomy. The pathological diagnosis showed clear cell carcinoma of the right ovary which arose from the MCT with malignant transformation pT2aNXM1. Although the patient underwent chemotherapy, she died after 17 months.

**Conclusion:**

This case is histologically characteristic of the proof of transition from simple squamous epithelium via simple glandular epithelium to papillary change with atypia. This is the first case report of unaccompanied clear cell carcinoma arising from MCT reported in English literatures.

## Background

Mature cystic teratomas (MCTs) are the most common germ cell tumors of the ovary, and malignant transformation of MCTs has been found in 1–2% of cases [1]. Seventy-five percent of these transformations are squamous cell carcinoma, with the other types being exceptionally rare [2]. We report the first known case of ovarian clear cell carcinoma arising from MCT.

## Case report

**Fig. 1:**
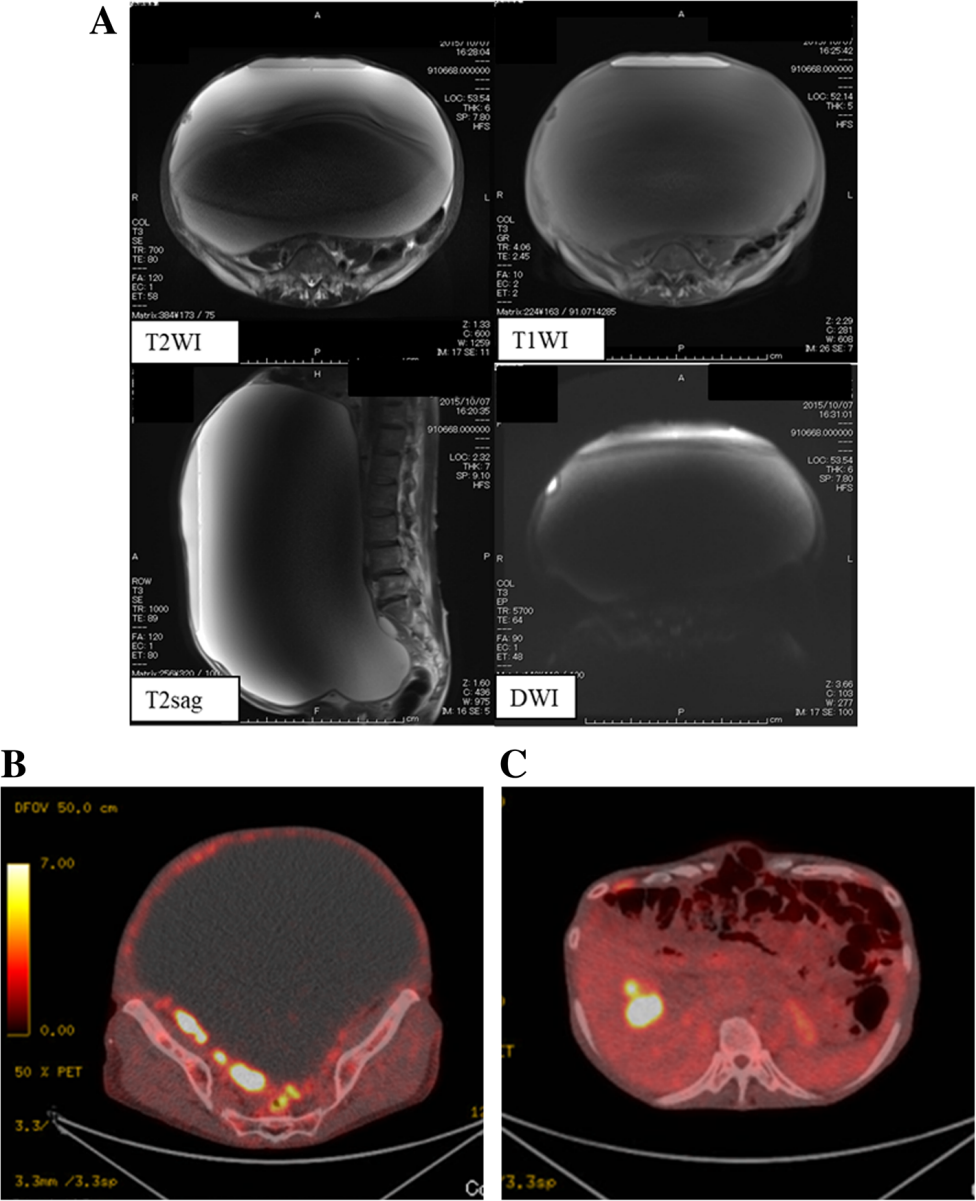
**a**. MRI findings: Horizontal T2-weighted and T1-weighted MR imaging demonstrated a water component which showed a drop in signal intensity with fat. **b**. FDG-PET showed FDG uptake in the pelvic tumor (SUVmax = 22.9). **c**. The hepatic nodule also showed FDG uptake (SUVmax = 13.7), which was suggestive of metastases

A 71-year old female, gravida 2, para 2 presented with abdominal distention which had worsened over the previous year. Her past medical history was appendicitis, and an appendectomy was performed in her thirties. Her past family history was unremarkable. A pelvic examination identified a very large mass in both hypochondrium which was hardly movable. Pelvic and abdominal ultrasonography showed a huge cystic mass with a solid component. Serum tumor marker levels were carcinoembryonic antigen (CEA): 2.0 ng/mL (normal < 37.0), CA19–9: 459.2 U/mL (normal < 37.0), SCC: 18.9 ng/mL (normal < 1.5), and CA125: 329.9 U/mL (normal < 35.0). Other blood examination results were unremarkable. A pelvic MRI showed a huge cystic mass with a nodular component which was enhanced and under diffused in diffusion weighted image (Fig. 1a). Computed tomography (CT) showed a 3 cm mass in the liver, and fluorodeoxyglucose-positron emission tomography (FDG-PET) showed FDG uptake not only in the pelvic tumor (SUVmax = 22.9) but also in the hepatic nodule (SUVmax = 13.7), thus suggesting metastases (Fig. 1b, c).

**Fig. 2:**
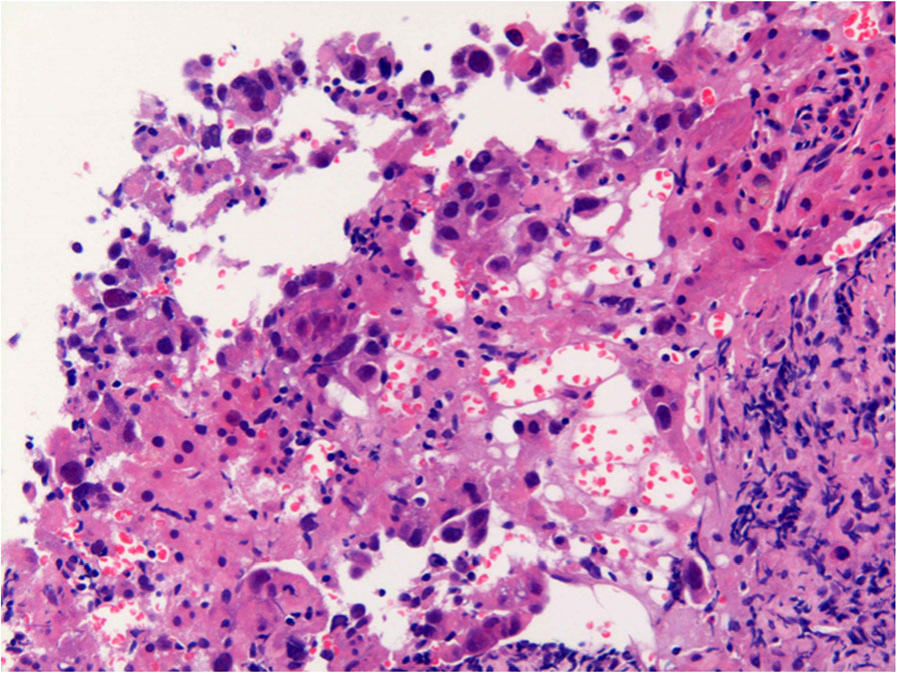
Histological microphotograph of liver biopsy (HE: × 400)

Ovarian cancer and liver metastasis was suspected on these data, and a subsequent percutaneous liver biopsy was performed. The pathology showed metastatic cells in normal hepatocytes, and a diagnosis of poorly differentiated carcinoma was made (Fig. 2). Immunohistochemical staining showed that p40, p63, and hepatocytes were all negative, thus denying primary hepatocellular carcinoma. Therefore, a clinical diagnosis of ovarian cancer stage IVB with malignant transformation of the MCT was made.

**Fig. 3:**
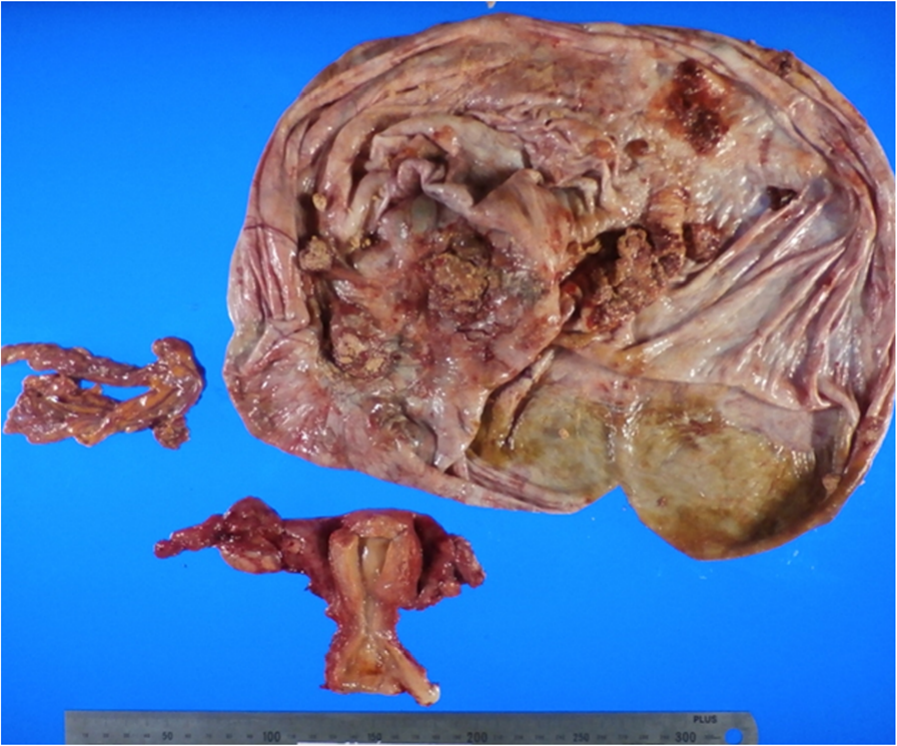
Resected specimen which includes the uterus, bilateral ovaries and omentum

A total abdominal hysterectomy, bilateral salpingo-oophorectomy and partial omentectomy was performed. The left ovary was enlarged (about 300 mm) and filled with 13,000 ml of yellowish fluid. Adhesion was not particular, and the uterus and right ovary appeared to have no remarkable changes (Fig. 3). The cytology of ascites was negative. The patient’s postoperative course was uneventful.

**Fig. 4:**
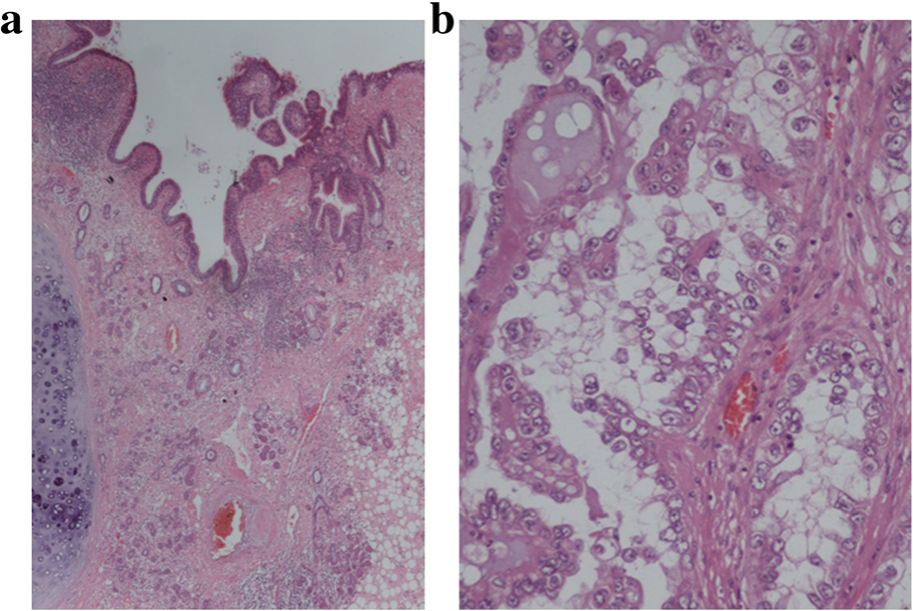
**a**. Left ovary mainly consisted of MCT with squamous cell epithelium, cutaneous appendage and cartilaginous tissue which were without atypia. (HE: × 40). **b**. Some parts of the left ovary were composed of cells with clear cytoplasm and hobnail cells. The pattern may be glandular or papillary. (HE: × 100)

**Fig. 5:**
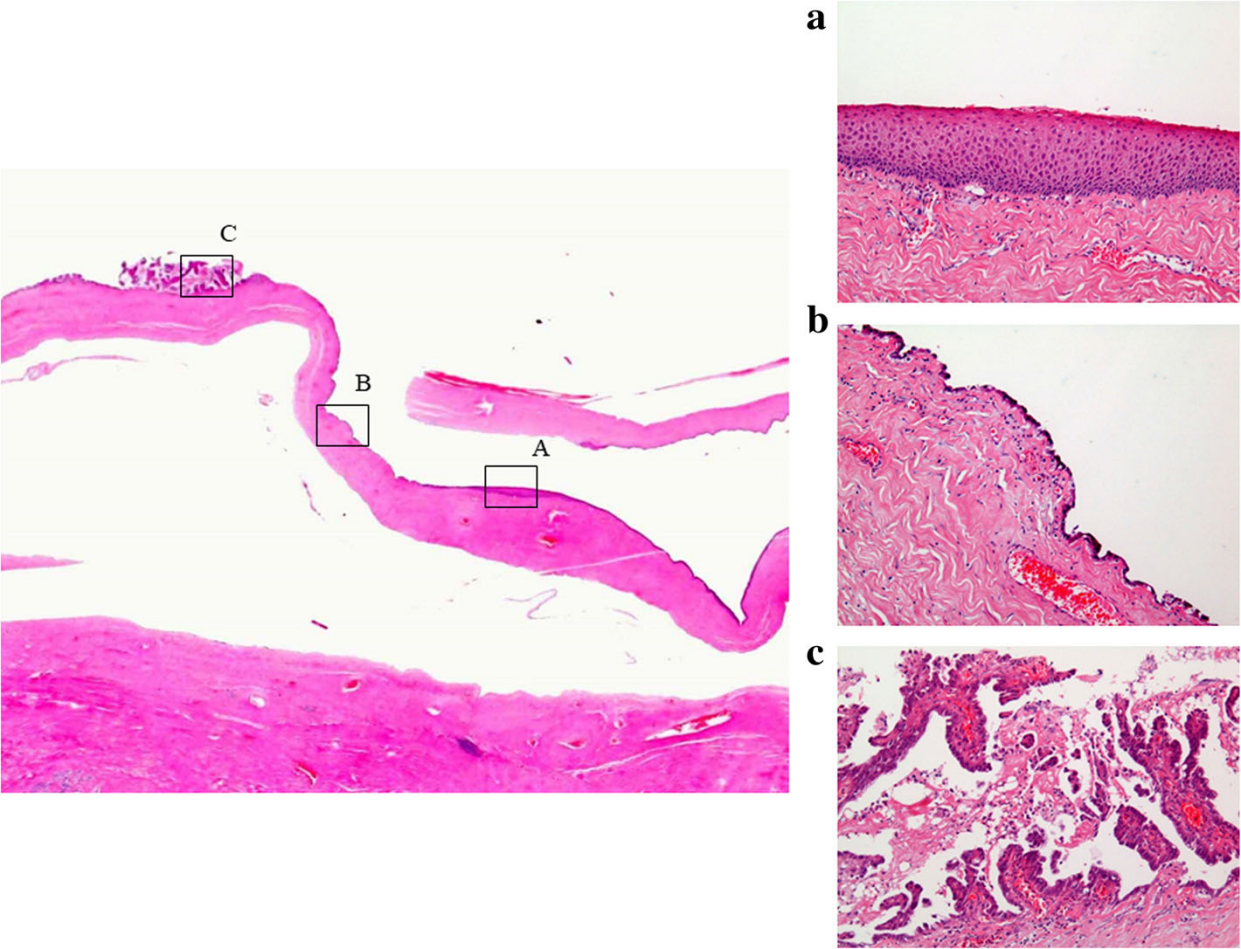
This case is histologically characteristic of the proof of transition from simple squamous epithelium (**a**), via simple glandular epithelium (**b**), to papillary change with atypia (**c**)

**Fig. 6:**
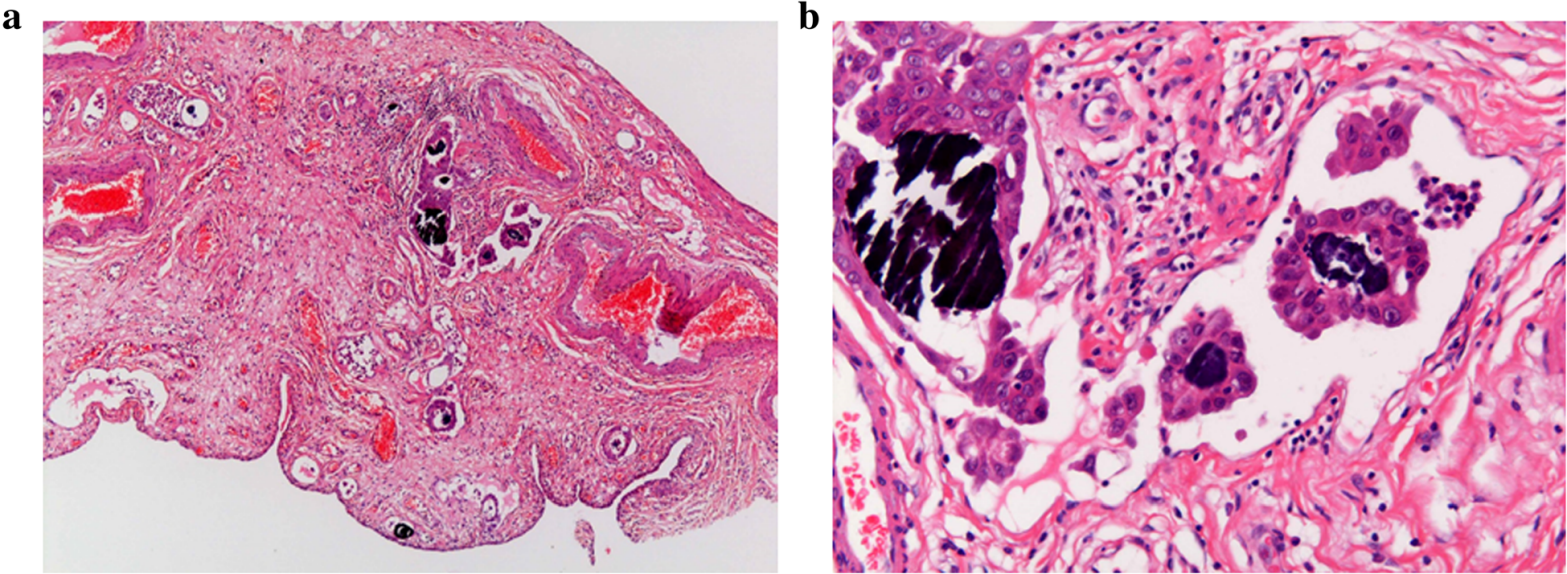
The left fimbria of the fallopian tube had a lymphovascular space of invasion of calcifying carcinoma cells. (**a**. HE: × 40, **b**. HE: × 200)

**Fig. 7:**
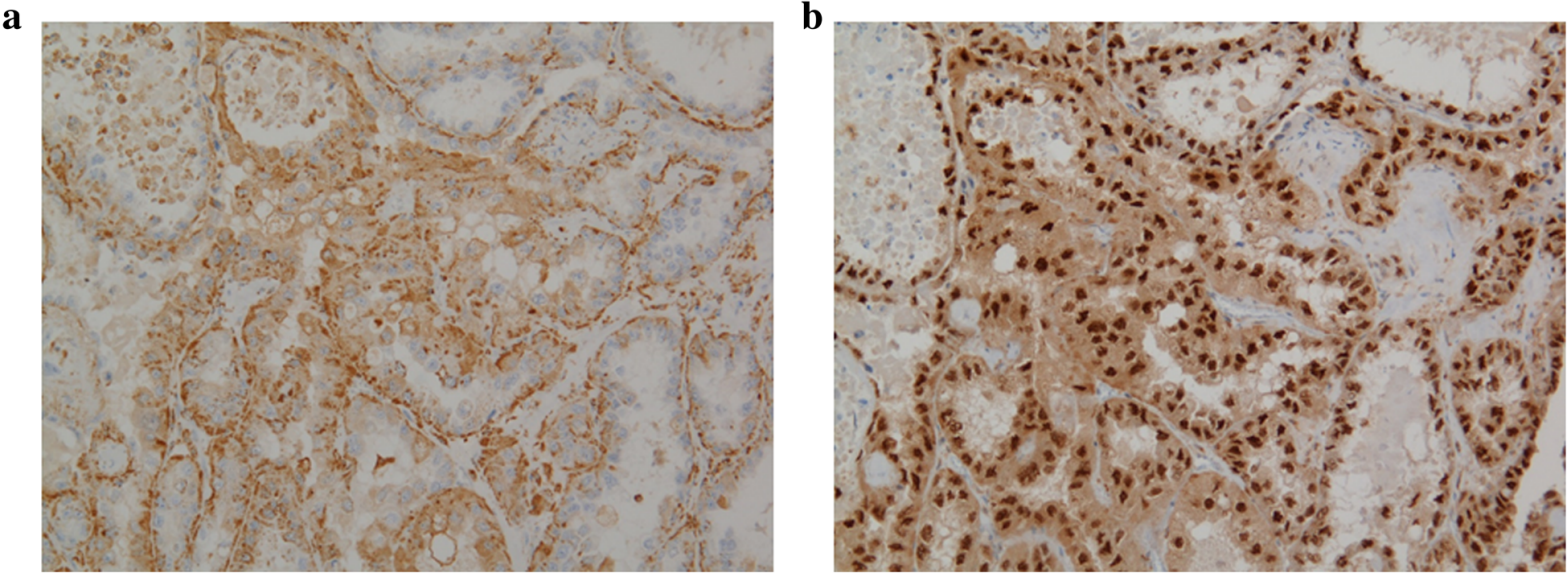
Immunohystochemical staining of the carcinomatous part. **a**. PAX-8 (HE: × 100). **b**. Napsin A (HE: × 100)

The pathological findings revealed that most of the ovary consisted of an MCT with squamous cell epithelium, cutaneous appendage and cartilaginous tissue (Fig. 4a); however, some parts consisted of papillary or glandular lesion with hobnail-like atypical cells (Fig. 4b). We made a diagnosis of left ovarian clear cell carcinoma and malignant transformation of MCT based upon the proof of transition from simple squamous epithelium via simple glandular epithelium to papillary change with atypia (Fig. 5). Histopathology showed no endometriosis in either ovary and, therefore, endometriosis-associated malignancy was denied. However, the uterus and omentum showed no signs of malignancy. The left fimbria of the fallopian tube had a lymphovascular space of invasion with calcifying carcinoma cells (Fig. 6a, b). However, the uterus and omentum showed no signs of malignancy. Immunohistochemical analysis of the left ovary indicated that AE1/AE3, CK7, PAX-8 and Napsin A were all positive, and that 34βE12 and P53 were slightly positive (Fig. 7a, b). Although CK20 and vimentin were negative, Ki-67 (MIB-1) index was 25% positive in the carcinomatous part. Moreover, PAS was positive in the stromal part, and PAS and ALB were both positive in some glandular lesions. This was classified as stage IVB (pT2aNXM1) according to the International Federation of Gynecology and Obstetrics (FIGO) 2014 classifications.

The patient subsequently underwent chemotherapy with 3 courses of paclitaxel and carboplatin. Afterward, CT scanning revealed a vanished hepatic metastasis and emerged swelling of the para-aorta lymph node. Although 3 courses of doxorubicin were administered, the para-aorta lymph node continued to enlarge. As chemotherapy was not effective due to her progressive disease, the patient began to receive the best possible supportive care. After 17 months from the operation, she passed away.

## Discussion

MCTs are common in women of childbearing age and account for 10–20% of all ovarian neoplasms. MCTs occur bilaterally in 10–17% of patients, and most patients with MCT are asymptomatic. However, they can develop pain and a sensation of abdominal fullness due to the mass [3]. MCTs might arise from germ cells by the failure of meiosis II or from a premeiotic cell in which meiosis I has failed [4].

A secondary malignant transformation of the various tissue components of MCT can occur, typically in postmenopausal women, and the prognosis is usually poor [3, 5–7]. The reported incidence of MCT is 1.2–14.2 cases per 100,000 per year, and the proportion of cases in which malignant transformation occurs is 0.17–2% [1]. Malignant transformation of MCT accounts for 1.5% of all ovarian malignant tumors and accounts for 36% of malignant ovarian germ cell tumors [8]. Hackethal et al. reported a mean age of 32 years and a mean tumor size of 64 mm for MCT, as well as 55 years and 148 mm for malignant transformation. Therefore, a patient age over 40 years and tumor size are predictors for secondary malignant transformation [3]. Mori et al. reported that the combination of the patient’s age (> 40 years) and serum SCC antigen level (> 2.5 ng/mL) was 77% sensitive and 96% specific for malignant transformation [9].

The most common form of malignant transformation of MCT is squamous cell carcinoma, occurring in about 75% of cases, and 7% are adenocarcinoma [2]. We reviewed literatures concerning epithelial ovarian cancer arising from MCT (excluding squamous cell carcinoma, mucinous carcinoma and thyroid carcinoma) from the last 20 years (Table 1) [10–23]. Table 1 shows that their adenocarcinomal histological subtypes arising from MCT are prostate-type 7adenocarcinoma [10], small cell carcinoma [11, 19], sebaceous carcinoma [12, 15, 18], undifferentiated carcinoma [13], carcinoid tumor [14, 20, 22], signet ring mucinous adenocarcinoma [16], large cell neuroendocrine carcinoma [17], urothelial carcinoma [21], and apocrine adenocarcinoma [23]. This case is the first case report of clear cell carcinoma arising from MCT reported in English literatures.

**Table 1:** Previous reports of epithelial ovarian cancer arising from MCT except squamous cell carcinoma and mucinous carcinoma in the last 20 years

case	Sourse	Age	Tumor size (cm)	Pathology
1	Lim SC, et al. (1998) [11]	28	28 × 21	small cell carcinoma
2	Ribeiro-Silva A, et al. (2003) [12]	63	15 × 12 × 10	sebaceous carcinoma
3	Kita N, et al. (2003) [13]	70	30 × 23 × 9	undifferentiated carcinoma
4	Guney N, (2009) [14]	54	8–9	carcinoid tumor
5	Venizelos ID, et al. (2009) [15]	74		sebaceous carcinoma
6	Boyd C, et al. (2012) [16]	35	5.2. × 2.0 × 4.1	pulmonary-type adenocarcinoma and signet ring mucinous adenocarcinoma
7	Miyamoto M, et al. (2012) [17]	69	15	large cell neuroendocrine carcinoma
8	An HJ, et al. (2013) [18]	69	22	sebaceous carcinoma
9	Rubio A, et al. (2015) [19]	37		small cell carcinoma
10	Tosuner Z, et al. (2015) [20]	75	8 × 7	carcinoid tumor
11	Chuang HY, et al. (2015) [21]	54	19 × 12 × 20	urothelial carcinoma
12	Kim JY. (2016) [22]	25	20.0 × 12.0 × 7.5	carcinoid tumor
13	Stanhiser J, et al. (2016) [10]	32	11	prostate-type adenocarcinoma
14	Holmes M, et al. (2018) [23]	32	4	apocrine adenocarcinoma
15	Present case	71	30	clear cell carcinoma

Due to the rarity of this tumor, adjuvant treatment has not been prospectively evaluated. As the selection of an effective treatment for clear cell carcinoma arising from MCT has not been established, we administered paclitaxel and carboplatin as the standard chemotherapy for ovarian carcinoma. However, clear cell carcinoma of the ovary is a quite unique ovarian tumor and shows resistance to platinum-based chemotherapy [24]. This case, which was clear cell carcinoma arising from MCT, also indicated a poor prognosis though chemotherapy. Consequently, further studies should evaluate other more effective agents for clear cell carcinoma arising from MCT.

When malignant transformation of MCT is diagnosed, it is very important to determine whether the clear cell carcinoma coexists incidentally or that the clear cell carcinoma arises from MCT. So far, three cases of the coexistence of MCT and clear cell carcinoma have been reported [25–27]. However, in those reports, it is not pathologically proven that clear cell carcinomas have arisen from MCT. This case is histologically characteristic of the proof of transition from simple squamous epithelium via simple glandular epithelium to papillary change with atypia. To our knowledge, this is the first case report of unaccompanied clear cell carcinoma arising from MCT reported in English literatures.

## Abbreviations

CEA: Carcinoembryonic antigen
FDG-PET: Fluorodeoxyglucose-positron emission tomography
FIGO: The International Federation of Gynecology and Obstetrics
MCTs: Mature cystic teratomas
